# Psychological and lifestyle predictors of paediatric sleep disturbances: a cross-sectional study

**DOI:** 10.1007/s00431-026-07254-1

**Published:** 2026-07-23

**Authors:** Agnese Suppiej, Elia Sigolotto, Cristina Malaventura

**Affiliations:** Paediatric Unit, University Hospital S. Anna, University of Ferrara, Ferrara, Italy

**Keywords:** Sleep disturbances, Child anxiety, Parental anxiety, Screen exposure, Sleep hygiene

## Abstract

Sleep disturbances are frequent in childhood and have been associated with emotional vulnerability and modifiable lifestyle behaviours. The relative and combined contributions of child anxiety, parental anxiety, and unstructured daily routines to paediatric sleep disturbances remain incompletely characterized. We conducted a cross-sectional study of 306 otherwise healthy children aged 3–14 years. Parents completed validated questionnaires assessing sleep disturbances, child anxiety, parental anxiety, and daily lifestyle habits, including evening screen exposure. Multivariable linear regression, mediation, and cluster analyses were performed to identify correlates of sleep disturbances and distinct profiles. Clinically significant sleep disturbances were identified in 39.2% of participants. Higher child trait anxiety was consistently associated with greater sleep disturbance severity (all *P* < .001). Evening screen exposure was independently associated with higher sleep disturbance scores (*P* < .05). Parental trait anxiety not directly associated with sleep disturbances both directly and indirectly through child trait anxiety, which mediated approximately 30% of the association (*P* < .001). Cluster analysis identified three distinct profiles, including two groups with comparable sleep disturbance severity but contrasting characteristics: one marked by high emotional vulnerability and one by predominant lifestyle dysregulation.

*Conclusion*: Sleep disturbances are strongly associated with trait anxiety and independently related to evening screen exposure, while parental anxiety appears to exert its effect both directly and, in part, indirectly through child anxiety. Similar sleep complaints may reflect different underlying emotional and lifestyle mechanisms. A multidimensional assessment integrating anxiety screening and evaluation of evening habits may improve early identification of at-risk children and support targeted preventive strategies within routine paediatric care.

**What is Known:**

• *Paediatric sleep disturbances are common and linked to psychological, family and lifestyle risk factors.*

• *Most studies examine these factors in isolation rather than within an integrated framework.*

**What is New:**

• *Child trait anxiety was the strongest correlate of sleep disturbances and partially mediated the effect of parental anxiety; evening screen exposure and later bedtime contributed independently.*

• *Children with similar sleep disturbance severity showed distinct emotional or lifestyle profiles, supporting multidimensional assessment.*

## Introduction

Sleep is a fundamental neurophysiological function that supports children’s brain development, emotional regulation, and overall psychological well being. Rather than being a passive state, sleep actively contributes to memory consolidation, emotional processing, and physical growth. Across childhood and adolescence, sleep patterns undergo dynamic changes shaped by neurobiological maturation and environmental influences [1, 2].

Sleep disturbances are frequently encountered in paediatric practice but are often underestimated during routine clinical evaluations [3].

Persistent sleep problems may contribute to, or represent, early manifestation of broader emotional vulnerability [4]. Evidence accumulated over the past two decades points to a close and bidirectional relationship between sleep disturbances and anxiety in children. Elevated anxiety levels have been associated with difficulties initiating and maintaining sleep, nocturnal awakenings, and daytime sleepiness, while chronic sleep disruption may further impair emotional regulation [5, 6]. Trait anxiety has been associated with persistent sleep problems across developmental stages and is thought to reflect a relatively stable tendency toward heightened emotional reactivity [7]. Despite this evidence, anxiety is not routinely screened when children present with sleep complaints in paediatric settings.

The family environment represents another relevant context for children’s sleep health. Parental anxiety and stress may influence sleep through family emotional climate, modelling behaviours, and bedtime interactions. Previous studies have reported associations between parental anxiety and poorer child sleep quality [8]. Beyond this direct association, models of intergenerational transmission of anxiety propose that parental anxiety influences child emotional functioning through multiple pathways, including modelling of anxious behaviours, transfer of threat-related information, and parenting characterised by overcontrol or reduced autonomy granting [9–11]. Because child anxiety is itself a robust correlate of paediatric sleep disturbances [5–7], these models predict that the association between parental anxiety and child sleep difficulties should be at least partially mediated by child anxiety. We therefore specified a priori the hypothesis that child trait anxiety mediates the association between parental anxiety and child sleep disturbances.

Alongside emotional factors, daily lifestyle behaviours have gained increasing attention as determinants of paediatric sleep. Evening screen exposure, irregular bedtimes, reduced physical activity, and dietary habits have all been associated with circadian disruption and fragmented sleep [12–15]. In the post-pandemic period, the persistence of unstructured routines and increased digital media use has further contributed to sleep difficulties in children [16]. All these behaviours are clinically relevant because they are potentially modifiable through anticipatory guidance and family-centered counselling [17, 18].

Importantly, several of these behavioural and emotional variables are known to vary across developmental stages. Age-related differences in autonomy, technology use, physical activity, and emotional regulation may influence both exposure and vulnerability to sleep disturbances. Therefore, accounting for age and sex is essential when examining associations between psychological, behavioural, and sleep variables in paediatric samples.

Although psychological and lifestyle factors have each been examined in relation to paediatric sleep disturbances, few studies have addressed their combined contribution within a single analytical framework. A more integrated approach may help clinicians to determine whether children’s sleep difficulties primarily reflect emotional vulnerability or whether unstructured daily routines play a dominant role. Such distinctions are likely to have practical implications for personalized preventive strategies in paediatric care.

The present study aimed to examine the independent and combined contributions of child anxiety, parental anxiety, and lifestyle behaviours to sleep disturbances in a community sample of healthy children. We further tested the a priori hypothesis that child trait anxiety mediates the association between parental anxiety and child sleep disturbances. By integrating conventional statistical analyses with exploratory modelling approaches, we sought to identify clinically meaningful risk profiles that may support early identification and targeted prevention in paediatric practice.

## Methods

### Study design

This study employed a cross-sectional observational design to evaluate sleep quality, anxiety levels, and lifestyle factors at a single time point using validated instruments.

Inclusion criteria were age between 3 and 14 years and provision of informed consent by parents, who voluntarily and anonymously completed an online form. Exclusion criteria included acute or chronic paediatric disorders and incomplete forms.

### Data collection procedure

Data were collected through an online parent-reported form created using Google Forms, accessible via direct link or QR code. Parents were informed about the study by family paediatricians and hospital paediatricians during well-child visits. Following verbal agreement, they received the link or QR code to access the survey.

Participation was voluntary and anonymous, and completion required approximately 15 min.

### Assessment instruments

The form included the following validated tools: the Sleep Disturbance Scale for Children (SDSC) to assess sleep quality and disorders [19, 20]; the State-Trait Anxiety Inventory (STAI) to assess parental anxiety; and the State-Trait Anxiety Inventory for Children (STAI-C) to assess child anxiety [21]. In addition, a custom section was developed to investigate lifestyle and environmental factors potentially affecting sleep hygiene.

### Sleep disturbance scale for children (SDSC)

The SDSC is a 26-item, 5-point Likert scale designed to assess overall sleep quality and six specific domains of sleep disturbance in children. The total score ranges from 26 to 130, with a score ≥ 39 considered indicative of potential sleep disorders. The SDSC evaluates the following domains: Disorders of Initiating and Maintaining Sleep (DIMS), Sleep Breathing Disorders (SBD), Disorders of Arousal (DA), Sleep–Wake Transition Disorders (SWTD), Disorders of Excessive Somnolence (DOES), and Sleep Hyperhidrosis (SHY). In the original validation study, the SDSC showed good internal consistency (Cronbach’s α = 0.79 in healthy controls; α = 0.71 in children with sleep disorders) and adequate test–retest reliability (r = 0.71) [19]. A Cronbach’s α of 0.83 was reported for the Italian preschool validation [20].

### State-trait anxiety inventory (STAI/STAI-C)

The STAI, administered to parents, assesses adult anxiety using two subscales: State Anxiety (STAI-S), reflecting a transient emotional response to specific situations, and Trait Anxiety (STAI-T), representing a stable tendency to perceive situations as threatening. Each subscale consists of 20 items rated on a 4-point Likert scale (score range 20–80).

The STAI-C, adapted for children aged 3–14, contains similar subscales rated on 3-point Likert scales. A score ≥ 30 was used as a clinical reference threshold. The STAI has shown excellent internal consistency in the original manual, with median Cronbach’s α coefficients of 0.92 for the State scale and 0.90 for the Trait scale. The STAI-C has shown adequate internal consistency in the original manual, with Cronbach’s α coefficients ranging from 0.78 to 0.87 across state and trait scales and across sex [21, 22].

### Lifestyle and environmental factors

To investigate modifiable daily habits associated with paediatric sleep hygiene, a dedicated section assessed lifestyle and contextual variables. Parents reported children’s bedtime timing, timing of physical activity, evening screen exposure (TV, videogames, device use), dietary habits (including sugary drinks, sweets, chocolate, and carbohydrate intake), and environmental characteristics (residential area: noisy/traffic vs quiet).

These variables were selected based on prior literature linking behavioural routines and environmental factors to sleep quality and were included to capture modifiable determinants of paediatric sleep health [23–25].

### Data analysis strategy

Following data cleaning and recoding, all analyses were conducted using RStudio (version 2024.12.1). Descriptive statistics were used to summarise sample characteristics and study variables (means and standard deviations for continuous variables; frequencies and percentages for categorical variables). All main regression models were adjusted for age, sex, and residential area, given their potential influence on both behavioural and psychological variables.

Multiple linear regression models were used to examine associations with continuous sleep disturbance scores (SDSC total score). Logistic regression models were used to examine predictors of clinically significant sleep disturbances (SDSC ≥ 39), with results reported as odds ratios (ORs) and 95% confidence intervals (CIs).

Random forest regression models (randomForest package) were used as exploratory analyses to assess the relative importance of predictors of sleep disturbances. Because anxiety and lifestyle factors were represented by different numbers of variables (four anxiety measures and fourteen lifestyle variables), variable-level importance scores are not directly comparable across the two domains. To allow a more balanced comparison, variable importance was therefore evaluated at three complementary levels. First, an individual variable-level random forest was fitted including the four anxiety variables, the fourteen lifestyle variables, and age, sex, and residential area as predictors of the SDSC total score. Second, intercorrelations among the fourteen lifestyle variables were examined using Spearman correlations, and percent increase in mean squared error (%IncMSE) values from the variable-level random forest were aggregated into two broad domains corresponding to anxiety (four variables) and lifestyle (fourteen variables). Because variable importance scores in random forests are not strictly additive when predictors are intercorrelated, aggregated %IncMSE values were interpreted as a descriptive convergence check rather than as a formal domain-level estimate. Third, as a secondary exploratory step, lifestyle sub-domains were derived using hierarchical clustering on the distance matrix defined as one minus the absolute Spearman correlation, and %IncMSE values were aggregated within each empirically derived lifestyle sub-domain. Finally, as the primary domain-level analysis, a random forest was fitted using standardised domain scores obtained by averaging standardised variables within each domain, while retaining age, sex, and residential area as covariates. This approach reduces each domain or sub-domain to a single composite predictor, thereby providing a domain-level importance estimate that mitigates the interpretive asymmetry caused by the differing number of variables originally representing each domain.

Mediation analyses were conducted to examine whether the association between parental anxiety and sleep disturbances was indirectly associated through child anxiety. The hypothesis that child anxiety mediates the association between parental anxiety and child sleep disturbances was specified a priori and is grounded in models of intergenerational transmission of anxiety. All models were adjusted for age, sex, and residential area. Indirect and direct effects were estimated using 5000 bootstrap resamples. Given the cross-sectional design, mediation results were interpreted as reflecting indirect associations rather than causal effects.

Cluster analysis was performed using behavioural and psychological variables to identify groups of children with similar profiles. Differences in age, sex, and sleep outcomes across clusters were subsequently examined.

Internal consistency coefficients (Cronbach’s α) for the original validated scales could not be computed, as item-level data were not available at the time of analysis. No missing data were present for the main variables included in the analyses. All tests were two-sided, with *p* < 0.05 considered statistically significant.

## Results

### Sample characteristics

The final sample included 306 children aged 3 to 14 years (mean = 8.90, SD = 1.67), with a balanced sex distribution (49.3% male, 50.7% female). Clinical sleep disturbances (SDSC ≥ 39) were observed in 39.2% of the sample. Table 1 presents the descriptive characteristics of the study sample.

**Table 1:** Descriptive characteristics of the study sample (*N* = 306)

Variable	Value
Age, mean (SD)	8.90 (1.67)
**Sex, n (%)**	
Female	155 (50.7)
Male	151 (49.3)
**Residential area, n (%)**	
Noisy/Traffic	237 (77.5)
Quiet	69 (22.5)
**SDSC total score, mean (SD)**	38.55 (9.25)
SDSC ≥ 39, n (%)	120 (39.2)
**Parental anxiety**	
STAI-State, mean (SD)	38.59 (8.09)
STAI-Trait, mean (SD)	42.04 (9.07)
**Child anxiety**	
STAI-C State, mean (SD)	28.27 (4.23)
STAI-C Trait, mean (SD)	30.09 (6.16)

### Anxiety and sleep disturbances

Descriptive analyses indicated moderate levels of parental and child anxiety across the sample. Both child state and trait anxiety showed positive associations with sleep disturbance scores, with stronger associations observed for child trait anxiety.

### Regression analyses

All regression models included age, sex, and residential area as covariates.

In adjusted linear regression analyses (controlling for age, sex, and residential area), higher child trait anxiety (β = 0.51, *p* < 0.001), parental trait anxiety (β = 0.14, *p* = 0.016), later bedtime (β = 2.48, *p* < 0.001), and evening television use (β = 1.28, *p* = 0.016) were significantly associated with higher SDSC scores. Residential area (noisy/traffic vs quiet) was also associated with higher SDSC scores (β = 3.80, *p* = 0.018).

Logistic regression analyses confirmed these findings. Child trait anxiety (OR = 1.13, 95% CI 1.07–1.18), parental trait anxiety (OR = 1.04, 95% CI 1.00–1.08), evening television use (OR = 1.36, 95% CI 1.01–1.84), and later bedtime (OR = 2.00, 95% CI 1.31–3.13) were associated with increased odds of clinically significant sleep disturbances. Residential area (noisy/traffic vs quiet) was also significant (OR = 2.59, 95% CI 1.07–6.42).

### Mediation analyses

Mediation analyses indicated a significant indirect association between parental trait anxiety and sleep disturbances through child trait anxiety (ACME = 0.076, 95% CI 0.029–0.130, *p* < 0.001), accounting for approximately 30% of the total association. A significant direct effect remained (ADE = 0.180,95% CI 0.073-0.291, *p* < 0.001), indicating partial mediation (Table 2).

**Table 2:** Mediation analysis examining indirect association between parental trait anxiety and sleep disturbances

Effect	Estimate	95% CI	p-value
ACME (indirect effect)	0.076	0.029–0.130	< 0.001
ADE (direct effect)	0.180	0.073–0.291	< 0.001
Total effect	0.256	0.147–0.372	< 0.001
Proportion mediated	29.7%	12.4% – 56.8%	< 0.001

*ACME*, average causal mediation effect; *ADE*, average direct effect. Estimates were derived from mediation models adjusted for age, sex, and residential area, with 95% confidence intervals obtained using 5,000 bootstrap resamples. Given the cross-sectional design, findings should be interpreted as reflecting indirect statistical associations rather than causal pathways

### Random forest analyses

Random forest analyses were conducted on the full set of original predictors, comprising four anxiety variables and fourteen lifestyle variables, while retaining age, sex, and residential area as covariates. The variable-level random forest explained 17.49% of the variance in SDSC total scores. At the individual predictor level, child trait anxiety showed the highest variable importance (%IncMSE = 17.38), followed by parental state anxiety (%IncMSE = 9.97), parental trait anxiety (%IncMSE = 9.50), bedtime timing (%IncMSE = 4.91), and child state anxiety (%IncMSE = 4.73).

To address the potential interpretive asymmetry arising from the different number of predictors used to represent anxiety and lifestyle factors, %IncMSE values from the original variable-level random forest were aggregated at the broadest domain level. Specifically, the four anxiety variables were combined into a single anxiety domain, whereas the fourteen lifestyle variables were combined into a lifestyle domain. Mean %IncMSE was substantially higher for the anxiety domain than for the lifestyle domain, 10.4 versus 1.55, respectively. Summed %IncMSE values showed the same pattern, with a higher value for the anxiety domain than for the lifestyle domain, 41.6 versus 21.7, respectively. These summed values are reported descriptively and should be interpreted with caution, as random forest variable importance scores are not strictly additive, particularly when predictors are intercorrelated.

**Fig. 1:**
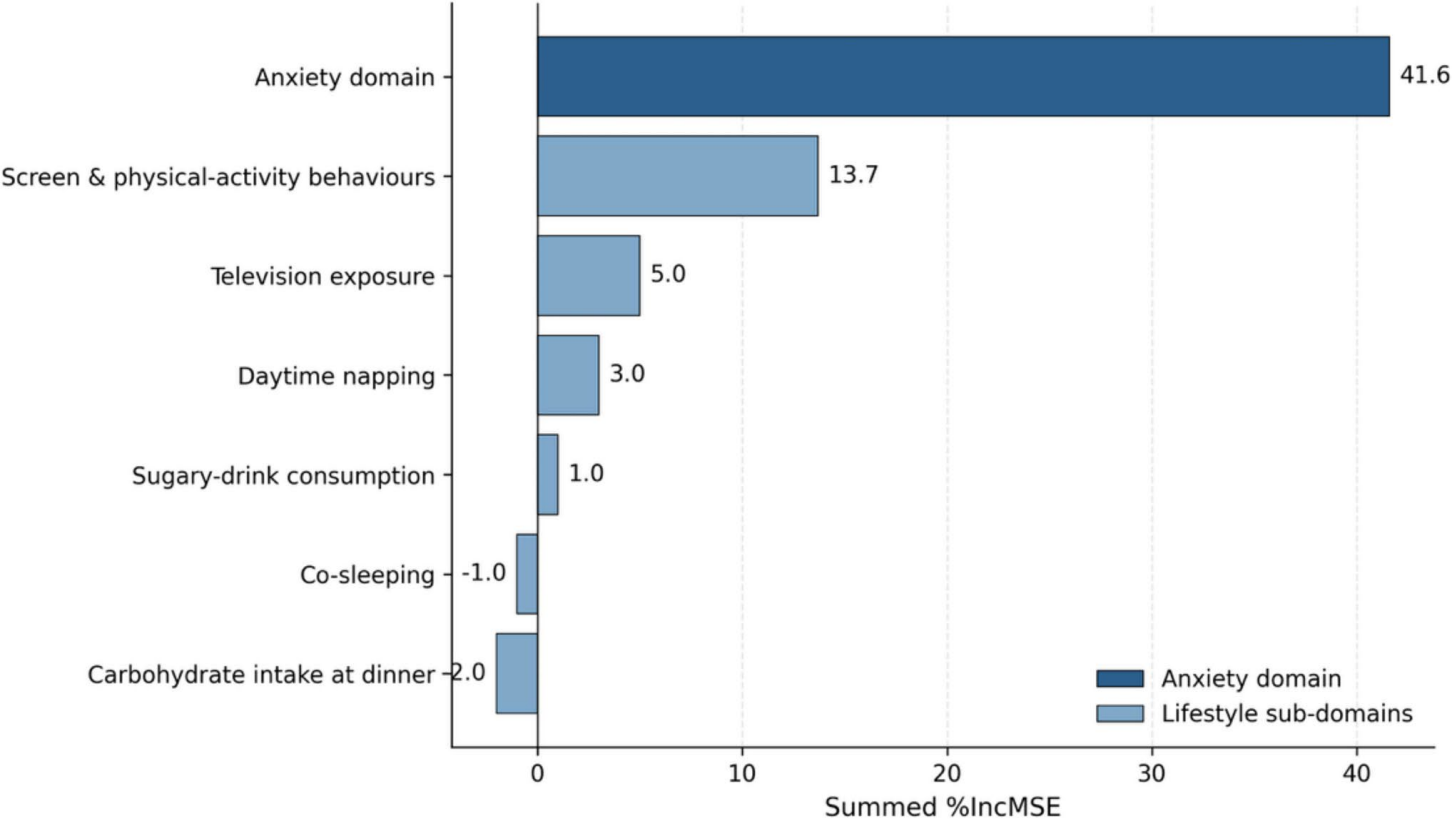
Aggregated variable-level random forest importance by anxiety domain and empirically derived lifestyle sub-domains. Bars represent the sum of percent increase in mean squared error (%IncMSE) values from the variable-level random forest, aggregated within the anxiety domain (four variables, dark blue bar) and within each of the six empirically derived lifestyle sub-domains (light blue bars). Higher values indicate greater contribution to the prediction of SDSC total scores. The anxiety domain shows substantially greater overall importance than any individual lifestyle sub-domain. Negative values reflect predictors whose permutation did not increase the mean squared error of the model. Abbreviations: SDSC, Sleep Disturbance Scale for Children; %IncMSE, percent increase in mean squared error. **Alt Text:** A horizontal bar graph ranks predictor domains for child sleep disturbances by their summed percent increase in mean squared error (%IncMSE) from the original variable-level random forest. The anxiety domain is the strongest contributor (summed %IncMSE = 41.6), followed by the screen and physical-activity behaviours sub-domain (13.7), television exposure (5.31), daytime napping (3.29), and sugary-drink consumption (1.15). Co-sleeping (− 0.72) and carbohydrate intake at dinner (− 1.11) show negative values, indicating that their permutation did not increase model error in this variable-importance metric

**Fig. 2:**
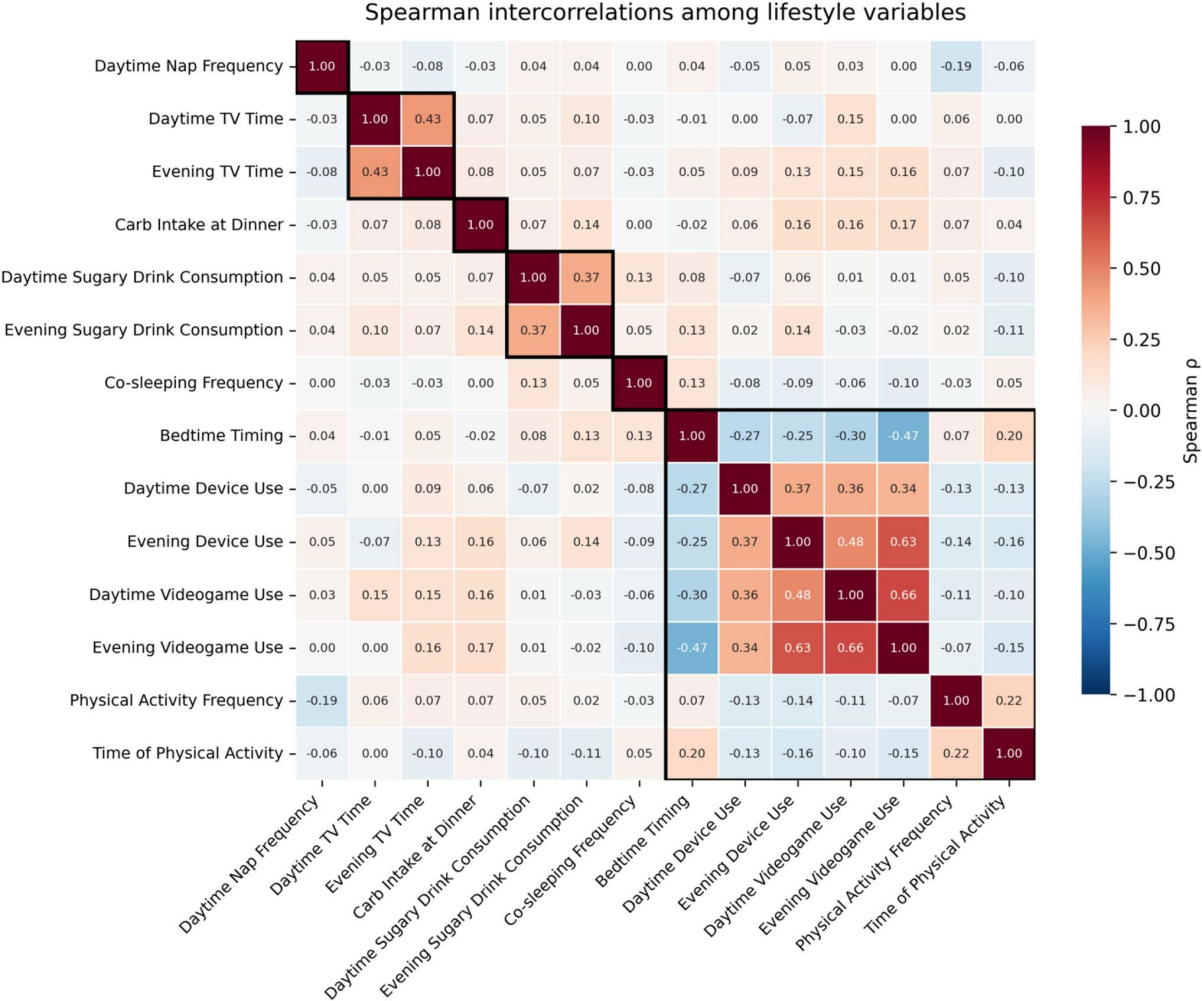
Spearman intercorrelation matrix among lifestyle variables. Heatmap displaying Spearman correlation coefficients among the fourteen lifestyle variables included in the random forest analyses. Variables are ordered according to hierarchical clustering, and black rectangles indicate the six empirically derived lifestyle sub-domains. Red shades indicate positive correlations and blue shades indicate negative correlations. **Alt Text:** A 14-by-14 heatmap displays Spearman correlation coefficients among lifestyle variables, with a divergent red-blue colour scale (red for positive, blue for negative correlations). Variables are ordered by hierarchical clustering, and six empirical sub-domains are outlined by black rectangles along the diagonal. The largest cluster (bottom-right) groups bedtime timing, daytime and evening device use, daytime and evening videogame use, and physical activity variables, with strong positive correlations among device and videogame variables (ρ between 0.34 and 0.66) and a negative correlation between bedtime timing and evening videogame use (ρ =  − 0.47). Smaller clusters include daytime and evening TV time (ρ = 0.43) and daytime and evening sugary-drink consumption (ρ = 0.37). Most other intercorrelations are weak (|ρ|< 0.20)

To further characterise the internal structure of the lifestyle domain, Spearman intercorrelations among the fourteen lifestyle variables were examined and used to derive empirical lifestyle sub-domains through hierarchical clustering. This procedure identified six lifestyle sub-domains, broadly corresponding to: (1) screen and physical-activity behaviours, including bedtime timing, physical activity timing and frequency, daytime and evening device use, and daytime and evening videogame use; (2) sugary-drink consumption, including daytime and evening intake; (3) television exposure, including daytime and evening television time; (4) daytime napping; (5) carbohydrate intake at dinner; and (6) co-sleeping. When variable importance scores from the original random forest were aggregated within these empirically derived sub-domains, no lifestyle sub-domain approached the importance of the anxiety domain. The largest lifestyle sub-domain was screen and physical-activity behaviours, with a summed %IncMSE of 13.7. Aggregated variable-level importance values are shown in Fig. 1, and the Spearman correlation matrix among lifestyle variables is shown in Fig. 2.

As the primary domain-level analysis, a separate random forest was fitted using standardised composite scores for the anxiety domain and for each of the six empirically derived lifestyle sub-domains, again retaining age, sex, and residential area as covariates. By reducing each domain or sub-domain to a single composite predictor, this analysis reduced the influence of differences in the number of variables originally representing anxiety and lifestyle factors. These domain-level estimates were obtained from a separate random forest model based on composite scores and are therefore not numerically comparable with the summed importance values shown in Fig. 1, which are reported as a descriptive convergence check. The domain-level model explained 12.39% of the variance in SDSC total scores. The anxiety domain emerged as the most important predictor (%IncMSE = 25.77), with a markedly higher importance value than the largest lifestyle sub-domain, screen and physical-activity behaviours (%IncMSE = 5.48), and the sugary-drink consumption sub-domain (%IncMSE = 4.17). This pattern converged with the descriptive aggregation of variable-level importance scores and supports the interpretation that the predominance of anxiety-related predictors was not merely an artefact of the smaller number of anxiety variables relative to lifestyle indicators.

### Cluster analysis

Cluster analysis identified three distinct profiles. Significant differences in sleep disturbance scores were observed across clusters (Kruskal–Wallis *p* < 0.001). Age also differed significantly between clusters (*p* < 0.001), whereas sex distribution did not differ significantly (*p* = 0.323).

The identified clusters broadly reflected: (1) a profile characterized by higher anxiety and poorer sleep outcomes, (2) a profile with higher behavioural dysregulation and intermediate sleep disturbance, and (3) a profile with lower anxiety, more regulated behaviours, and better sleep quality.

**Fig. 3:**
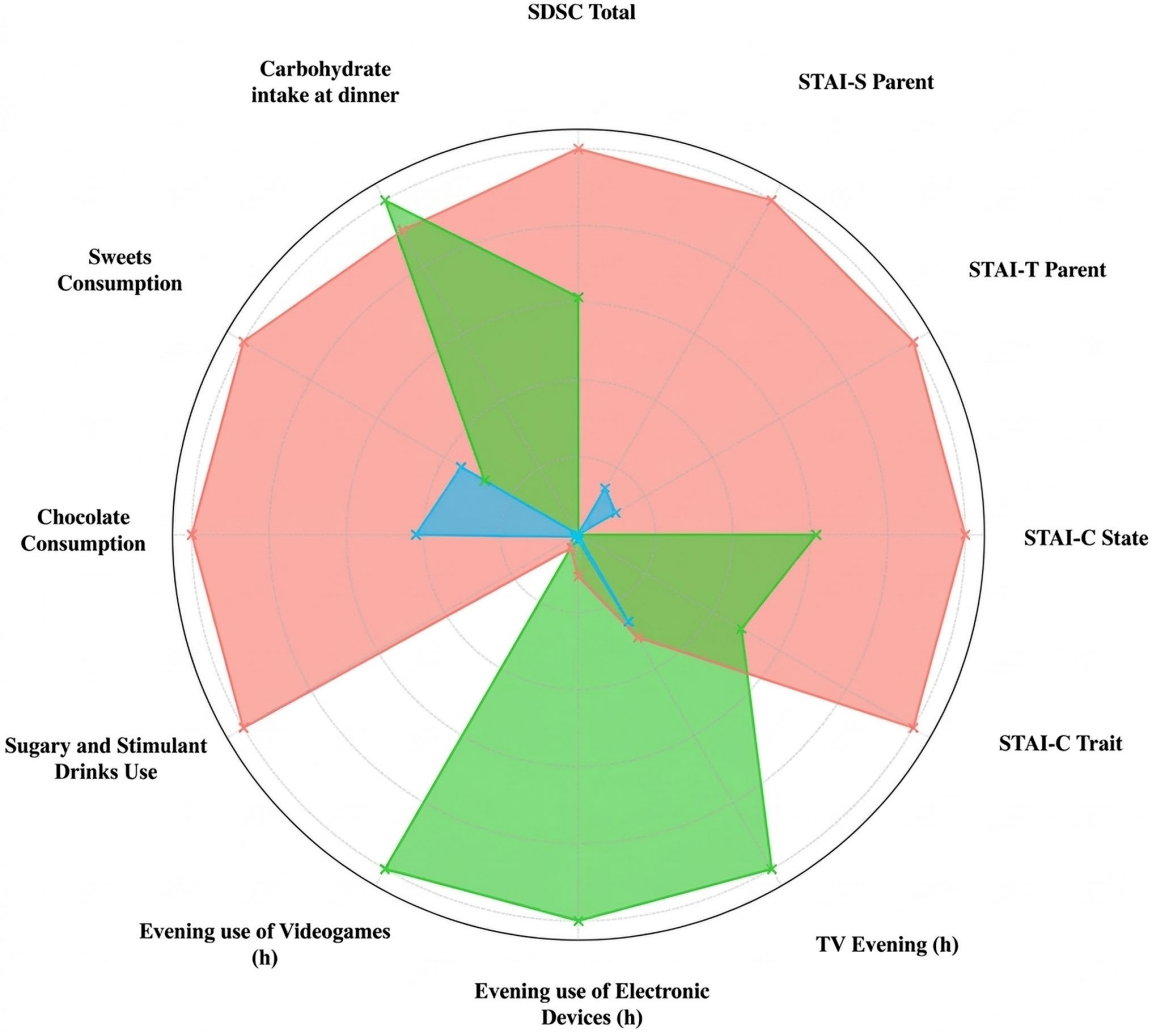
Radar chart illustrating the three behavioural-emotional profiles identified by cluster analysis. Data are normalized: values represent the percentage relative to the maximum score observed across the three clusters for each variable (100 = maximum value). The shading intensity differentiates the clusters: Red area represents Cluster 1 (Emotionally Vulnerable); Green area represents Cluster 2 (Lifestyle Dysregulated); Blue area represents Cluster 3 (Regulated). Abbreviations: SDSC, Sleep Disturbance Scale for Children; STAI, State-Trait Anxiety Inventory. **Alt Text:** A radar chart displays three behavioural-emotional profiles identified by k-means cluster analysis across multiple normalised sleep, anxiety, and lifestyle variables. The Emotionally Vulnerable profile (medium grey area) extends outward on the anxiety and sleep disturbance axes; the Lifestyle Dysregulated profile (dark grey area) extends outward on screen-time and gaming axes; the Regulated profile (light grey area) remains close to the centre across all axes, indicating low values throughout

Results of Cluster Analysis (k-means clustering) identified three distinct profiles (Fig. 3; Table 3).

**Table 3:** Cluster analysis identifying behavioural-emotional profiles of paediatric sleep disturbances (mean ± SD)

Variable	Cluster 1: Emotionally Vulnerable Profile	Cluster 2: Lifestyle Dysregulated Profile	Cluster 3: Emotionally and Lifestyle Regulated Profile
SDSC Total Score	43.91 ± 9.97	40.53 ± 9.09	33.82 ± 5.61
Parental Anxiety – State	44.54 ± 7.22	33.84 ± 6.29	35.67 ± 6.46
Parental Anxiety – Trait	48.18 ± 8.00	37.96 ± 8.68	38.75 ± 7.35
Child Anxiety – State	30.52 ± 4.47	28.73 ± 3.21	26.40 ± 3.42
Child Anxiety – Trait	33.84 ± 6.03	29.57 ± 5.98	27.41 ± 4.72
Evening TV Time	0.60 ± 0.80	1.08 ± 1.48	0.55 ± 0.69
Evening Device Use	0.33 ± 0.64	2.86 ± 1.76	0.14 ± 0.37
Evening Videogame Time	0.24 ± 0.68	3.84 ± 0.66	0.10 ± 0.35
Sugary Drink Consumption	0.13 ± 0.36	0.00 ± 0.00	0.01 ± 0.12
Chocolate Consumption	0.54 ± 0.63	0.06 ± 0.24	0.23 ± 0.50
Sweet Consumption	0.45 ± 0.50	0.29 ± 0.46	0.32 ± 0.47
Carbohydrate Intake at Dinner	2.49 ± 1.09	2.61 ± 1.11	2.04 ± 1.06

Values are presented as mean ± SD for descriptive comparison of cluster profiles

Cluster analysis was exploratory and not used for hypothesis testing

## Discussion

In this community-based sample of children aged 3 to 14 years, sleep disturbances were common and consistently linked to both emotional vulnerability and aspects of children’s daily routines. Across all analytical approaches, emotional factors, particularly child trait anxiety, showed stronger and more consistent associations with sleep disturbances than lifestyle variables. These findings support the conceptualization of disordered sleep as an early clinical signal of emotional vulnerability rather than a purely behavioural complaint [5, 26].

Trait anxiety emerged as a more stable and clinically relevant correlate of sleep disturbance than state anxiety. While state anxiety reflects transient fluctuations in response to situational stressors, trait anxiety represents a more enduring predisposition toward heightened emotional reactivity. This distinction is clinically meaningful, as persistent emotional vulnerability may contribute to chronic difficulties in sleep initiation, maintenance, and daytime functioning. These findings are consistent with previous research linking stable anxiety traits to long-term sleep disturbances across development [7].

Parental anxiety showed both direct and indirect associations with children’s sleep disturbances, with a significant proportion of the association mediated by child anxiety. This pattern suggests that parental emotional distress may influence sleep partly through its impact on children’s emotional regulation. Such findings are consistent with models of intergenerational transmission of anxiety, in which parental affective states shape children’s emotional experiences over time [9, 10]. However, given the cross-sectional design, these findings should be interpreted as reflecting indirect associations rather than causal pathways.

Lifestyle behaviours also contributed to sleep disturbances, although their effect sizes were smaller compared to emotional factors. Evening television use and later bedtime were independently associated with poorer sleep outcomes, in line with evidence linking screen exposure and irregular routines to circadian disruption and cognitive overstimulation [27]. Although these effects were modest, their clinical relevance remains high, as lifestyle behaviours are modifiable and represent key targets for preventive interventions. Structured routines and reduced evening screen exposure may be particularly beneficial in children with elevated emotional vulnerability [16, 28, 29].

The relative contribution of emotional and behavioural factors was further explored using machine-learning approaches. While random forest analyses at the individual variable level identified anxiety-related measures as the most influential predictors, a potential interpretive limitation of variable-level importance scores arises from the fact that anxiety and lifestyle factors were represented by different numbers of variables. To address this, a domain-level random forest was fitted using composite scores that reduce each domain to a single predictor, an approach that is unaffected by the differing number of variables originally representing each domain. The anxiety domain emerged as the most important predictor, markedly higher than any lifestyle sub-domain. This finding converged with a descriptive aggregation of variable-level %IncMSE values across the two domains, although the latter should be interpreted cautiously given that importance scores are not strictly additive when predictors are intercorrelated. Together, these analyses suggest that the predominance of anxiety-related predictors does not merely reflect the smaller number of anxiety variables relative to lifestyle indicators, and they strengthen confidence in the interpretation that emotional vulnerability, and trait anxiety in particular, represents the most clinically meaningful correlate of paediatric sleep disturbances in this sample.

Cluster analysis provided additional insight by identifying distinct profiles of children characterized by different combinations of emotional and behavioural features. Notably, clusters differed significantly in both sleep disturbance severity and age, highlighting the importance of developmental factors in shaping sleep-related behaviours and vulnerabilities. In particular, the Lifestyle Dysregulated cluster, characterised by greater evening device and videogame use, comprised on average older children, consistent with the increased autonomy in media use that accompanies later childhood and early adolescence. As pubertal transitions are themselves associated with shifts in circadian preference and sleep architecture, the behavioural features of this cluster may partly co-occur with, rather than independently drive, age-related vulnerability to sleep disturbances. The inclusion of age as a covariate in all main regression models was intended to address this issue, but residual developmental confounding cannot be excluded. The presence of profiles with comparable sleep disturbance levels but differing underlying characteristics suggests that similar clinical presentations may arise from distinct pathways.

These findings have important clinical implications. Children whose sleep disturbances are primarily associated with emotional vulnerability may benefit from psychological interventions targeting anxiety and emotional regulation, whereas those with lifestyle-related sleep difficulties may respond more effectively to behavioural and routine-based interventions. Recognizing these distinct pathways may support more tailored and effective management strategies in paediatric practice.

### Limitations

Several limitations should be considered. First, the cross-sectional design precludes causal inferences. Second, socioeconomic and family-level variables, including parental education, household income, and number of siblings, were not collected as part of the study design and could therefore not be included as covariates; this represents a meaningful limitation, as such variables are plausible correlates of both anxiety and lifestyle behaviours and may confound the associations examined. Third, the use of parent-reported measures may introduce reporting bias. Fourth, internal consistency of the original validated scales (Cronbach’s α) could not be computed in our sample, as item-level data were not available at the time of analysis; however, all instruments used in the study have demonstrated good internal consistency in their original validation studies (SDSC: α = 0.79–0.83; STAI: α = 0.90–0.92; STAI-C: α = 0.78–0.87). Finally, the exploratory nature of the machine-learning and clustering analyses should be considered when interpreting the findings.

## Conclusions

Paediatric sleep disturbances appear to arise from the interaction of emotional vulnerability, family context, and daily routines. Trait anxiety, in particular, may represent an early and clinically meaningful marker of risk that can be identified in routine paediatric settings. Integrating brief emotional screening with information on healthy evening routines may support earlier identification of at-risk children.

Moreover, the identification of distinct profiles of sleep disturbance suggests that similar clinical presentations may arise from different underlying pathways, supporting the need for personalized preventive strategies. Viewing sleep complaints not only as isolated symptoms but also as indicators of broader emotional and behavioural vulnerability may enhance the role of paediatric care in promoting children’s long-term mental and developmental health.

Future research should adopt longitudinal designs to clarify the temporal relationships between anxiety, lifestyle behaviours, and sleep disturbances, and to better understand their role in long-term mental health outcomes.

## Data Availability

The datasets generated and/or analysed during the current study are available from the corresponding author on reasonable request.

## List of abbreviations

CI: Confidence interval
DA: Disorders of Arousal
DIMS: Disorders of Initiating and Maintaining Sleep
DOES: Disorders of Excessive Somnolence
OR: Odds ratio
SBD: Sleep Breathing Disorders
SDSC: Sleep Disturbance Scale for Children
SHY: Sleep Hyperhidrosis
STAI: State-Trait Anxiety Inventory
STAI-C: State-Trait Anxiety Inventory for Children
STAI-S: State Anxiety Subscale
STAI-T: Trait Anxiety Subscale
SWTD: Sleep-Wake Transition Disorders

## Declarations

### Use of AI-assisted technologies

During the preparation of this work, the authors used ChatGPT (OpenAI) solely to improve language, readability, and grammatical accuracy. After using this tool, the authors reviewed and edited the content as needed and take full responsibility for the content of the publication.
